# Subtotal colectomy with antiperistaltic cecosigmoidal anastomosis may be another suitable option for slow transit constipation: Experiences from Chinese people

**DOI:** 10.1097/MD.0000000000019065

**Published:** 2020-02-14

**Authors:** Yong-Ping Yang, Jian Shi, Ze-Yun Zhao, Ling-Yun Yu, Tong-Jun Liu

**Affiliations:** a Department of General Surgery, The Second Hospital of Jilin University; b Department of Ear Nose and Throat Surgery, The First Hospital of Jilin University, Changchun, China.

**Keywords:** antiperistaltic cecosigmoidal anastomosis, slow transit constipation, subtotal colectomy

## Abstract

The objective of this paper is to demonstrate, considering the experiences from Chinese people, if slow transit constipation (STC) can be accurately diagnosed, choosing patients qualifying for surgery, subtotal colectomy with antiperistaltic cecosigmoidal anastomosis (STC-Anti-CSA) may come with more acceptable short and long-term outcomes.

A retrospective study was performed at a department of colorectal and anal surgery. A cohort of 29 patients were coming with up to 5 years’ follow-up care, who were in a diverse range of age, BMI, laxative medicine histories, including both males and females. Pre-surgery work-up strictly followed a protocol designed to rule out the patients who were not suitable for surgery treatments. Classification of STC was followed after diagnosis. STC-Anti-CSA was performed in all cases. The operative time, blood loss, average post-operative length of stay (LOS), frequency of BMs, stool consistency and patients satisfaction, by using Wexner constipation score (WCS), numerical rating scale (NRS), and abdominal bloating score (ABS), over the study period were recorded.

In this study, there were 14 males and 15 females, with mean age 51, and BMI from 20.14 to 31.62 kg/m^2^. The period of laxative medicine history was 4.8 years (2–13 years). The mean operative time was 152 ± 34 min, and the mean perioperative blood loss was 123 ± 51 mL. Average post-operative LOS (LOS) was 8 days. There were no severe post-operative complications, peri-operative mortality, anastomotic leaks, or revisions of the original surgery. Initial post-operative BMs averaged 6 times/day. During the period of 1 month to 12 months follow-up care, BMs fell down to 2 or 3 times/day. By the 1st to 3rd year follow-up care, BMs averaged 5 to 7 times/week. However, from the 4th year to 5th year, constipations recurred somehow. However, most patients were satisfied with their bowel patterns.

STC-Anti-CSA can receive acceptable postoperative outcomes as long as the patients can be accurately diagnosed and classified as severe STC. Among the surgical procedures for STC, this procedure may be another suitable option, especially for Chinese people.

## 1 Introduction

Approximately 15% of the adult Chinese population are suffered from chronic constipation.^[1]^ A subgroup of this disease has a more severe form of constipation called slow transit constipation (STC), which is characterized by a longer period of time without any defecation, with or without progressively worsening abdominal distention and even pain.^[2]^ Normally such patients would all have a laxative taking-history, including osmotic laxatives, prescription medications, supplemental medicine of higher fiber intake. Some patients have surpassed dietary changes but no obvious improvements. In the initial period of STC, patients can have a limited benefit from laxatives, fiber intaking, and dietary changes.^[3,4]^ However, usually over years and even decades, these treatments would become less and less efficient, and less benefits can be received from such kinds of medicines, and there usually coming with progressive worsening abdominal distention and less BMs. To defecate and relieve this constipation, patients have to resort to a higher dose of laxative, which can cause a higher volume capacity of colon such as dilation, elongation, and redundancy.^[5]^ Such changes of colon reversely can cause more severe constipation, which may require acute hospitalization for abdominal distention and obstructive symptoms, such as nausea, vomiting, and pain. Unfortunately, even after acute hospital treatment, including a placement of a nasogastric tube for gastric decompressing, enema from anus, some STC patients still need an acute emergent surgery of colostomy due to some of colon wall necrosis from heavy stool burden.

To avoid such severe outcomes happened, along with selecting a surgical timepoint which can receive a better postoperative recover, as a surgeon, how to accurately diagnose and classify the STC severity becomes a top thing. A tailored selection of surgery process before life-threatening complications occurring is indispensable. The objectives of this study are:

1. to classify the STC patients who need a surgical treatment and then
2. to demonstrate the outcomes and benefits of a subtotal colectomy with antiperistaltic cecosigmoidal anastomosis procedure, comparing with other surgical treatments, among Chinese people.

## 2 Methods

A written informed consent was obtained from the patient, and institutional Ethics Committee of the Second Hospital of Jilin University approved this study.

From April 2010 to May 2014, in the Second Hospital of Jilin University, 29 patients were selected and followed up until now. For the safety and effectiveness of the treatment plan chosen, before the surgeries involved, the surgical indications are strictly followed as: all the patients to receive a surgery should be qualified for

1. the Rome III diagnosis criteria for constipation;
2. at least two positive Sitz Mark Colon Transit Studies to confirm the exist of STC;
3. laxative medicine-taking history is more than 2 years;
4. Wexner constipation score (WCS, on a scale of 0–30 in which a higher score means more severe constipation, normally the score of a healthy person is <8) is more than 15;
5. age range is from 18 years old to 70 years old;
6. surgical involvement is strongly desired by the patient.

Meanwhile, there are also exclusion criteria:

1. some sever comorbid conditions, such as liver dysfunction, and aspiration dysfunction, not suitable for a surgical procedure;
2. evidence of psychological symptoms or history of mental illness;
3. diagnosis of outlet obstruction;
4. combined with life-threatening diseases;
5. evidence of organic colon disease.

Finally, all the patients underwent surgery.

**Figure 1:**
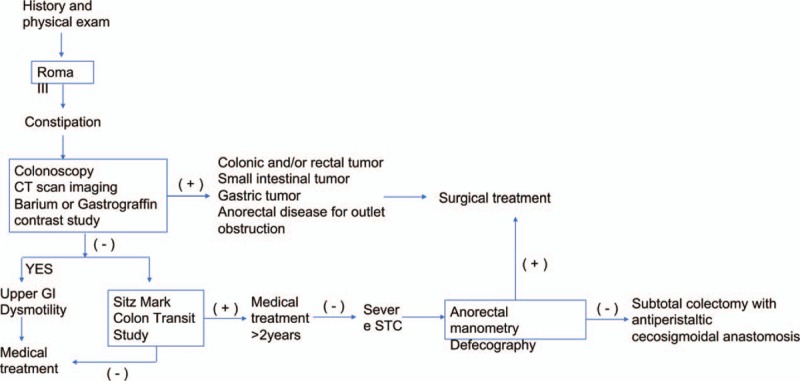
Algorithm of workup of slow transit constipation in this study.

The patients’ characteristics are listed in Table 1. In addition, all of them were scored according to a WCS. Twenty-eight patients in 29 had a score above 20, 1 patient had a score of 18.4. All study patients were having a traditional medicine-taking history, which were given by a physician, including laxative, stimulant medications for at least 2 years. Two of them were taken enema treatment occasionally by doctors. The diagram and protocol of work-up followed is presented in Figure 1. All the 29 patients, pre-operationally, underwent a colonoscopy examination, barium contrast enema, anal rectal pressure test, and anal examination, due to rule out colon obstructive constipation by organic etiologies,^[6,7]^ which was also mentioned above in the exclusion criteria. These examinations were necessary and indispensable, because if ruled out, the patients meant to should been underwent another different surgical procedure involving. A key diagnostic maneuver used is Sitz Mark Colon Transit Study, which is not only been used to diagnose but also to classify the severe degree of STC. All the candidates in this study showed a value of 5 or more out of 20 radio-opaque markers present on abdominal X-ray on the 5th day after the initial, which were considered to be a positive result.

**Table 1:**
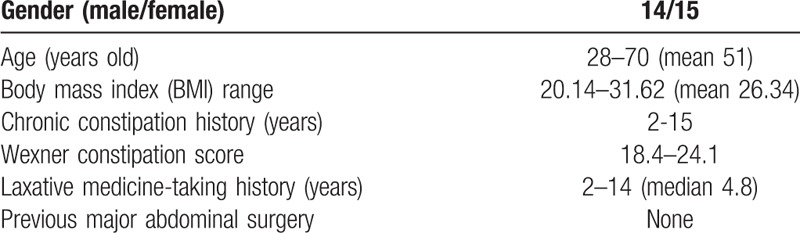
Patient characteristics.

Subtotal colectomy with antiperistaltic cecosigmoidal anastomosis was performed on all the patients. The ascending colon was dissected at around 10 cm from ileocecal valve. At the meantime, the ileocolic mesentery, where the ileocecal vessels were, and cecum were carefully preserved in every case. The distal margin was at sigmoid colon, around 10 to 15 cm up from peritoneal reflection. The distance from peritoneal reflection reserved depended on the individual situation about the colon margin vessel supplying and the length of ileocolic and sigmoid colonic mesentery. Close the left ascending colon. An end to end anastomosis was performed by a circular stapler approximating the cecal stump (at the site of the appendiceal orifice) to the sigmoid colonic stump. Considering the blood supply, rotation of the ileocolic mesentery should be avoided when creating the anastomosis. During hospitalization, the operative time, blood loss, complications, if occurred, and post-operative length of stay (LOS) were recorded. Post-discharge from the hospital, frequency of BMs, stool consistency, and patients satisfaction at the time set point of 1 week post-surgery, 1 month, 3 months, 6 months, 1 year, 2 years, 3 years, 4 years, 5 years were recorded. Long-time complications should also be recorded, such as abdominal pain and distention, constipation, incisional hernia, and anastomosis leak, if there happened. All the data were collected through out telephone calls after discharge from hospital.

Two figures were designed to evaluate the outcome of treatment pre- and post-operation. One of them is an efficacy endpoint which has recorded the overall symptom improvement score given by the patients at the timepoint of the 6th month, the 1st year, the 2nd year, the 3rd year, the 4th year, and the 5th year post-operation, with the standard following statement: “The treatment helped to improve my bowel problems,” using a 5-point Likert Scale (0: strongly disagree, 1: disagree, 2: neither agree nor disagree, 3: agree, and 4: strongly agree). Meanwhile, another efficacy endpoint was also applied to each patient. In that figure, improvements of severity of individual constipation symptom were assessed by 10-cm Visual Analogue Scale ("nothing” to “extremely intense”). Furthermore, a thorough table was used to assess to functional recovery compared at different time points (1 week pre-operation, 6 months post-operation, 1 year post-operation, 3 years post-operation, and 5 years post-operation), by using the scores of WCS, abdominal pain intensity assessed by the numerical rating scale (NRS, ranging from 0 to 10, in which 0–3 represents mild pain, 4–6 represents moderate tolerable pain, and 7–10 represents severe pain), and the abdominal bloating score (ABS, ranging from 0 to 4, in which 0 means absent, 1 means occasionally, 2 means sometimes, 3 means most of the time, and 4 means all the time).

## 3 Results

As shown in Table 1, clinical characteristics in this cohort consist of 14 males and 15 females, with age from 28 years old to 70 years old. The BMI range is from 20.14 to 31.62 kg/m^2^, with a mean of 26.18 kg/m^2^, which suggested obese for Chinese people. All the candidates involved in this study had a chronic constipation history over 2 years (2 years to 15 years), with the WCS ranging from 18.4 to 24.1. All the patients had had a laxative-taking history through the STC, from 2 years to 14 years. No patients had received a major abdominal surgery before this one. The operative data and hospital LOS have been shown in Table 2. Operative time was 152 ± 34 min, and estimated blood loss was 123 ± 51 mL. Average length of hospital stay (LOS) was 8 days.

**Table 2:**
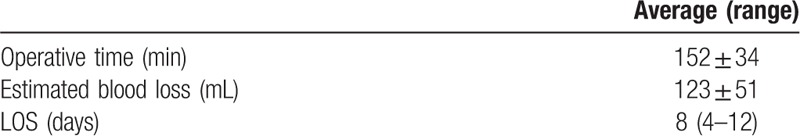
Operative data and hospital length of stay (LOS).

Surgery related complications are shown in Table 3. Perioperative complications (within 1 month) were limited to medicine-controlled abdominal pain (24 patients in 29) and abdominal distention (14 patients in 29). There were no anastomotic leak, revisions of the original surgery and mortality happened as long-term complications. However, there were 4 patients with medicine-controlled abdominal pain, 3 patients with abdominal distention, and 1 patient with incisional hernia occurring 1 month after surgery.

**Table 3:**
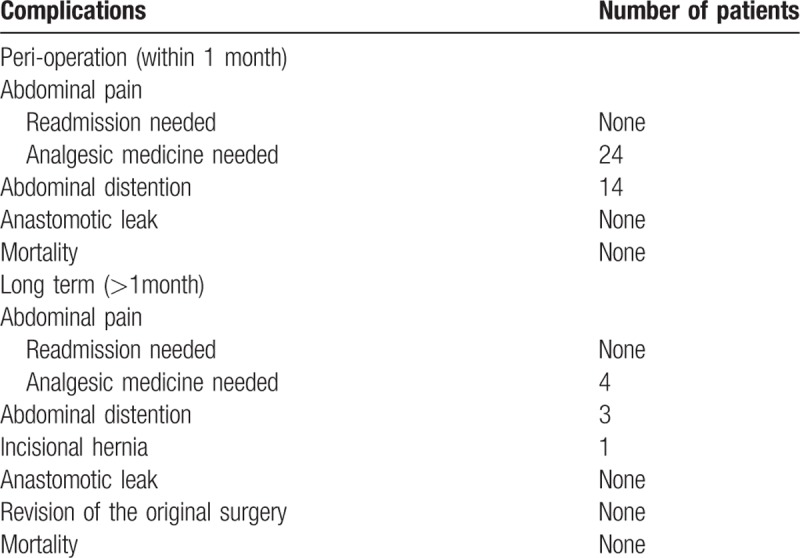
Post-operation complications.

**Figure 2:**
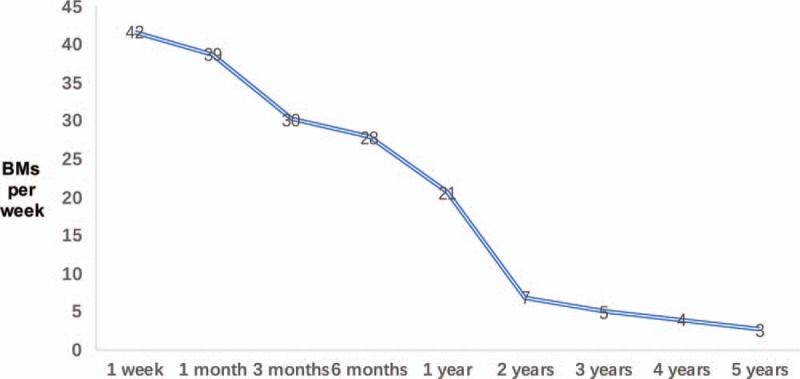
Frequency of bowel movements (BMs) vs time after surgery.

Post-operative frequency of BMs is tabulated in Figure 2. At the 1st week, BMs averaged 7 times/day. However, the frequency fell down to 3 times/week rapidly at around 1 year. This descending trend was consistent throughout this 5 years. The BMs were 2 to 4 per week at 4 to 5 years after the surgery. When 5 years after the surgery, constipation recurred for some of the patients, who turned to an osmotic laxative for assisting. Even though, all the patients were satisfied with their bowel patterns, comparing with that before such surgeries.

**Figure 3:**
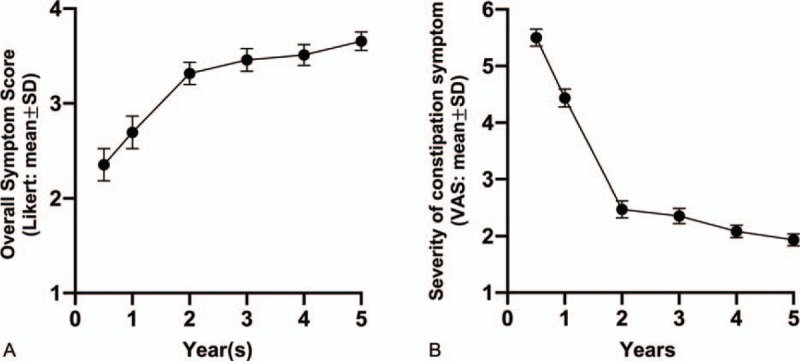
Assessment of pre- and post-operation by time points. (A) Evaluation of overall symptom improvement score at the timepoint of the 6th month, the 1st year, the 2nd year, the 3rd year, the 4th year, and the 5th year post-operation by using a 5-point Likert Scale. (B) Evaluation of improvements of severity of constipation symptom at the timepoint of the 6th month, the 1st year, the 2nd year, the 3rd year, the 4th year, and the 5th year post-operation by using a 10-cm Visual Analogue Scale (VAS).

The improvements of overall STC symptom assessment post-operationally are shown in Figure 3A, by using a 5-point Likert Scale, meanwhile, the post-operational evaluation of severity of constipation are tabulated in Figure 3B by the patients with a 10-cm VAS. In Figure 3A, the overall symptom were improved by time, and significantly got better at the 2nd year after the surgery. The detail scores of Figure 3A were 2.35 ± 0.65 at the 6th month post-operation, 2.69 ± 0.17 at the 1st year post-operation, 3.32 ± 0.12 at the 2nd year post-operation, 3.46 ± 0.12 at the 3rd year post-operation, 3.51 ± 0.11 at the 4th post-operation, and 3.66 ± 0.10 at the 5th post-operation. Interestingly, the trend of recovery was also found in Figure 3B, in which severity of constipation was largely improved at the 2nd year around after the surgery. The data of Figure 3B were 5.50 ± 0.15 at the 6th month post-operation, 4.43 ± 0.16 at the 1st year post-operation, 2.47 ± 0.15 at the 2nd year post-operation, 2.36 ± 0.13 at the 3rd year post-operation, 2.09 ± 0.11 at the 4th post-operation, and 1.93 ± 0.10 at the 5th post-operation. However, in both Figure 3A and B, the recovery of STC were tending to become steady, without getting much better or worse from the 4th year on after the surgery. Even constipation in some of the patients recurred somehow at the 5 years after the surgery, who had to turn to laxative resolved. All the patients were satisfied with the outcome of the surgery comparing with the symptoms pre-operation.

Pre- and post-operational functional recovery results were shown in Table 4, measured by WCS, ABS, and NRS. The score of WCS was significantly improved at the 3rd year, as same as that of ABS and NRS. No obvious changes were found at the 5th year after the surgery any more.

**Table 4:**
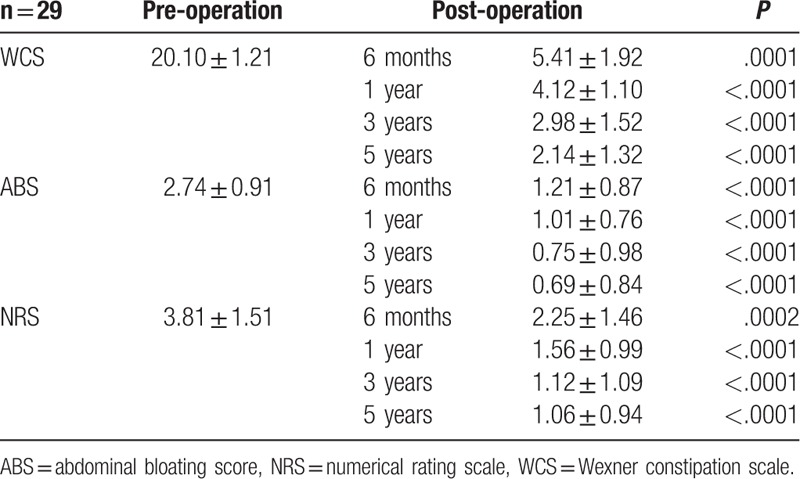
Pre- and post-operational functional recovery results (mean ± SD).

## 4 Discussion

The STC has a high morbidity in China.^[1]^ Although the pathogenesis of STC is still unknown quite clearly, increasing doctors tend to believe that STC is a functional disorder, charactering by continuing colonic smooth muscle activity decreasing, intrinsic colonic nerves decreasing, and the number of cells of Cajal reducing.^[8]^ Until now, no effective non-surgery approaches have been recommended toward the entire nervous system degeneration. Therefore, the colon with STC adapts to becoming a non-functional pouch or reservoir. At this view, a surgery involvement would become the only approach eventually. However, the timepoint of surgery involvement is top crucial, as is shown in Figure 1. Besides the type of surgical approach chosen on patients, who is qualified to receive the surgery and when to perform this surgery is equally important related to the outcomes of treatment.

In terms of treatments for STC, surgery is a common and usually the final approach, especially for the patients with poor responses to conservative treatment, such as laxative, and diet changes.^[9]^ Nowadays, two surgical approaches are acceptable worldwidely, one is total colectomy with ileorectal anastomosis, the other is subtotal colectomy with ceco-rectal anastomosis. The former is widely used especially by the surgeons from Europe and the United States, because of its high cure rate and low recurrence. However, the patients receiving this such surgery are tend to suffer from refractory diarrhea, abdominal pain, and distention.^[10–13]^ The latter preserves the ileocecal valve, which can remain the pressure difference between ilium and cecum. The patients who receive this term of treatment can have a lower morbidity of refractory diarrhea, which would definitely bring with a higher quality of post-surgery life.^[14]^

Our institute performed some subtotal colectomies with ceco-rectal anastomosis on the patients of STC. The patients were found that also tend to suffer from unacceptable increased BMs. We compared the anatomy between the Chinese people and European people, and then came to a hypothesis that Chinese patients having more refractory diarrhea rates due to the reason that the Chinese people had a longer sigmoid colon and shorter rectum than the Europeans. So when the sigmoid colon was removed from abdomen, there were not enough organ to deal with the water ingredients in stools for Chinese, which would definitely lead to a diarrhea. Due to this fact, our institute began to perform the surgery of subtotal colectomy with antiperistaltic cecosigmoidal anastomosis, which preserved more length of colon than that of the ceco-rectal anastomosis.

In fact, the type of surgery for the STC to perform, such as whether total or subtotal colectomy, whether ileocecal valve sparing or not, whether isoperistaltic or antiperistaltic cecorectal anastomosis, have been discussed for many years. For every type of surgery, there were successful cases with high cure rate and more BMs than before, meanwhile usually with several failure cases with recurrence of constipation. What should be more meaningful to analysis is the reasons of causing the failure cases than others. In our opinion, the key point of surgery efficacy is different surgical design depending upon different individual situation. Considering this viewpoint, we highlight the type of surgery for the Chinese STC patients is subtotal colectomy with antiperistaltic cecosigmoidal anastomosis, which is different from other surgeries, due to the reasons as follows:

1. the Chinese people tend to have a longer sigmoid colon than European and American people, so such procedure can bring an easier process for surgeons which is not require a rotation of the ileocecal junction and could avoid causing blood vessel torsion, and is more easier to anastomosis than ileo- or ceco-rectal anastomosis. Furthermore, the longer sigmoid colon preserved can lead less tension around anastomosis, which can decrease the incidence of anastomosis leak.
2. A longer sigmoid colon preserved can absorb more water ingredients from stool, which can be helpful to alleviate morbidity of refractory diarrhea after the surgery, and the bacterium in the sigmoid colon can also be preserved, which can provide the benefit to maintain the hemostasis of micro-environment around.
3. The preservation of the ileocecal valve during this surgery has been proved to have an advantage accepted widely. With the function of ileocecal valve, the food ingredients can stay a longer time in the terminal ileum, as a result, electrolyte, bile salt, and vitamin B12 can have a better absorption.^[15]^ An additional benefit is the ileocecal valve can provide a sphincter-like function, which can decrease the pressure with ileum distention, meanwhile maintain the pressure in cecum and ascending colon. That sphincter-like function of ileocecal valve can prevent the back flow of bacterial content from the cecum to ileum.^[16]^
4. The antiperitaltic anastomosis can make a pouch-like organ, which can delay the intestinal content emptying, avoiding the refractory diarrhea post-surgery.

As shown in Figure 3A, the overall symptoms of the patients who had received a surgery procedure of subtotal colectomy with antiperistaltic cecosigmoidal anastomosis have been improved in some degree. Severity of individual constipation symptom, which was evaluated by a 10-cm VAS, also showed a significant improvement in Figure 3B. Interestingly, the data in this study group were changed largely at around 2nd year post-operationally. Lately, a thorough investment of the patients’ satisfaction were assessed which was tabulated in Table 4. The data of WCS, ABS and NRS were all improved post-operationally, as the same trend as that shown in the Figure 3.

As shown in Table 5, we have compared the post-surgery effective rates with other Chinese STC surgery groups by using the WCS. We have found that our group had rather acceptable better results. Xu has a score of 4.9 ± 2.0 in 39 cases of subtotal colectomy with antiperistaltic cecorectal anastomosis, a score of 3.3 ± 1.7 in 39 cases of total colectomy with ileorectal anastomosis.^[17]^ Wang has a score of 5.4 ± 1.6 in 41 cases of total colectomy with ileorectal anastomosis.^[18]^ Zhang has a score of 3.2 ± 1.8 in 23 cases of total colectomy with ileorectal anastomosis, a score of 3.7 ± 1.6 in 22 cases of subtotal colectomy with ileosigmoidal anastomosis.^[19]^ Yu has a score of 5.0 ± 1.0 in 31 cases of open-abdominal both total colectomy with ileorectal anastomosis and subtotal colectomy with antiperistaltic cecorectal anastomosis.^[20]^ Song has a score of 6.11 ± 2.09 in 41 cases of subtotal colectomy with cecorectal anastomosis, a score of 4.51 ± 1.8 in 41 cases of total colectomy with ileosigmoidal anastomosis.^[21]^ Finally, our study has score of 4.12 ± 1.10 in 29 cases with subtotal colectomy with antiperistaltic cecosigmoidal anastomosis, which has a better result than most surgery groups. Among the antiperistaltic anastomosis groups, our study has the best score result, comparing with the Xu's, Yu's, and Song's group. It shows us that sigmoid colon reserved does not lead to constipation as long as the length of sigmoid colon remaining is proper. In our study, the distal margin of sigmoid colon is around 10 to 15 cm up from peritoneal reflection, which means the location of anastomosis is very near to the junction of sigmoid colon and rectum. That anastomosis can have the benefits of avoiding the refractory diarrhea along with the ileocecal valve existing, preserving the function of colon as much as possible, and making the surgery more easier than the ileorectal anastomosis.

**Table 5:**
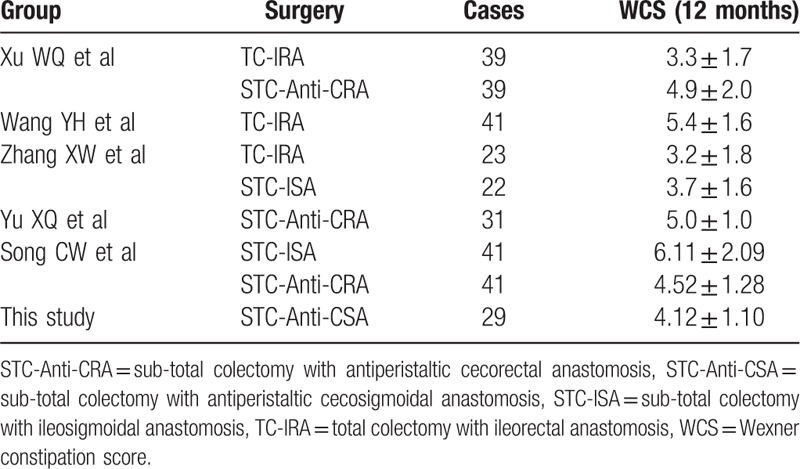
Wexner constipation score (WCS) at 12 months post-operation in different surgery groups.

## 5 Conclusion

This study demonstrates that, following algorithm of workup of STC, the surgical indications criteria and excluding criteria, it is feasible to perform this surgery of subtotal colectomy with antiperistaltic cecosigmoidal anastomosis with optimal short- and long-term results for severe STC as compared with other Chinese surgical groups, who performed subtotal and/or total colectomy. Meanwhile implications of this study long-term results suggest that, considering the constipation would recur ultimately, a minimally invasive surgery could be recommended as a better choice, such as subtotal colectomy with antiperistaltic cecosigmoidal anastomosis, comparing with total colectomy. Finally, subtotal colectomy with antipeirstaltic cecosigmoidal anastomosis can be another suitable surgical procedure, especially for Chinese people.

## Acknowledgments

I would like to thank Dr. Jian Shi for assistance in preparation of the manuscript.

## Author contributions

**Conceptualization:** yongping yang, Tongjun Liu.

**Data curation:** yongping yang, Jian Shi, Zeyun Zhao, Lingyun Yu.

**Formal analysis:** yongping yang, Jian Shi, Zeyun Zhao.

**Methodology:** yongping yang, Jian Shi, Tongjun Liu.

**Resources:** Zeyun Zhao.

**Supervision:** Lingyun Yu, Tongjun Liu.

**Writing – original draft:** yongping yang, Lingyun Yu.

**Writing – review & editing:** Lingyun Yu, Tongjun Liu.
